# Whole exome sequencing identifies a mutation in thrombomodulin as the genetic cause of a suspected platelet disorder in a family with normal platelet function

**DOI:** 10.1080/09537104.2017.1283011

**Published:** 2017-08-18

**Authors:** Annabel Maclachlan, Gerry Dolan, Charlotte Grimley, Steve P. Watson, Neil V. Morgan, on behalf of the UK GAPP Study Group

**Affiliations:** ^a^ Institute of Cardiovascular Sciences, College of Medical and Dental Sciences, University of Birmingham, Birmingham, UK; ^b^ Nottingham Haemophilia Centre, Nottingham University Hospital, Nottingham, UK

**Keywords:** Genetics, inherited bleeding, next-generation sequencing, platelets, thrombomodulin

## Abstract

Here, we describe a mother and son with a lifelong bleeding tendency and posttraumatic bleeding who were recruited to the UK Genotyping and Phenotyping of Platelets (GAPP) study with a suspected platelet function disorder. However, despite a clinically significant bleeding score, both had normal platelet counts and normal platelet function. The patients’ blood was analyzed by light transmission aggregometry and genotyping by whole exome sequencing, as outlined by the GAPP study. Approximately 25 000 genetic variants were found for each patient as a result of sequencing and were filtered using a specialized bioinformatics pipeline. A heterozygous variant displaying autosomal dominant inheritance (c.1611 C>A) was found in the gene *THBD* which encodes the glycoprotein thrombomodulin. This sequence change results in a stop codon (p.Cys537Stop) and truncation of the protein and has been previously described in two other families with bleeding events which suggests it may be a recurrent mutation. In summary, this study shows that patients with a suspected platelet disorder but who present with a normal pattern of platelet aggregation should be investigated for defects in nonplatelet genes.

## Introduction

Inherited platelet disorders (IPDs) are a heterogeneous group of disorders associated with normal or reduced platelet counts and bleeding diatheses of varying severities [1,2]. The identification of the underlying cause of IPDs is clinically challenging, and, as a consequence, a DNA-based approach has a highly important role in the investigation of these patients [3]. The UK Genotyping and Phenotyping of Platelets (GAPP) study recruits patients with mild bleeding who have been clinically diagnosed with a suspected platelet function disorder. However, approximately 60% of patients recruited have no apparent platelet defect, despite having a clinically significant bleeding score. A mother and son were recruited to the GAPP study with a lifelong bleeding tendency, including posttraumatic bleeding, but presented with normal platelet and platelet-rich plasma (PRP) counts (4.2 × 10^8^/mL and 3 × 10^8^/mL, respectively), normal clotting factors, and normal platelet function. Factor IX genetics were also normal; however, prothrombin consumption index was abnormal. To study these patients further, we have used a forward genetic approach by implementing whole exome sequencing (WES) focusing on nonplatelet genes.

## Methods

The mother and son were evaluated, and a clinical history was taken including bleeding scores using the International Society of Haemostasis and Thrombosis bleeding assessment tool [4]. Blood was obtained from both patients, PRP was isolated, and platelet function was tested using lumiaggregometry. A dual Chrono-log 460VS lumiaggregometer (Chrono-log, LabMedics, UK) was used to test patient PRP using a range of concentrations of agonists: ADP (100 µM, 30 µM and 10 µM, 3 µM, 1 µM), adrenaline (30 µM and 10 µM), arachidonic acid (2.25 mM and 1.5 mM), U46619 (3 µM and 1 µM), PAR1 agonist (100 µM, 30 µM, and 10 µM), PAR4 agonist (500 µM, 250 µM, and 100 µM), collagen (3 µg/mL, 1 µg/mL, and 0.3 µg/mL), collagen-related peptide (CRP) (1 µg/mL), and ristocetin (1.25 mg/mL and 1.5 mg/mL). Platelet ATP secretion from dense granules was also measured using the luciferase assay and agonists: ADP (100 µM), adrenaline (30 µM), U46619 (3 µM), PAR1 agonist (100 µM), PAR4 agonist (500 µM), collagen (3 µg/mL), and CRP (1 µg/mL) [5].

WES was performed with a minimum of 1μg DNA extracted from peripheral blood. Enrichment of the coding regions and intron/exon boundaries was completed with the SureSelect Human All Exon 50 Mb kit (Agilent Technologies, UK) according to the manufacturer’s instructions and analyzed as previously described [6]. The novelty of filtered variants was elucidated by comparison to known variants present within dbSNP139, exome aggregation consortium (ExAC), the 1000 Genomes Project and EVS (evs.gs.washington.edu/EVS/), as well as the data generated from over 1200 exomes of in-house controls sequenced and analyzed using the same protocol [7]. Copy number variants were detected using ExomeDepth [8]. Variants were then called using a custom-made bioinformatics pipeline to determine candidate variants from the WES data as previously shown by the GAPP study [6]. Sanger sequencing was then performed on candidate variants to confirm the variants, thereby ruling out the possibility of false-positive results of WES.

## Results

**Figure 1.**
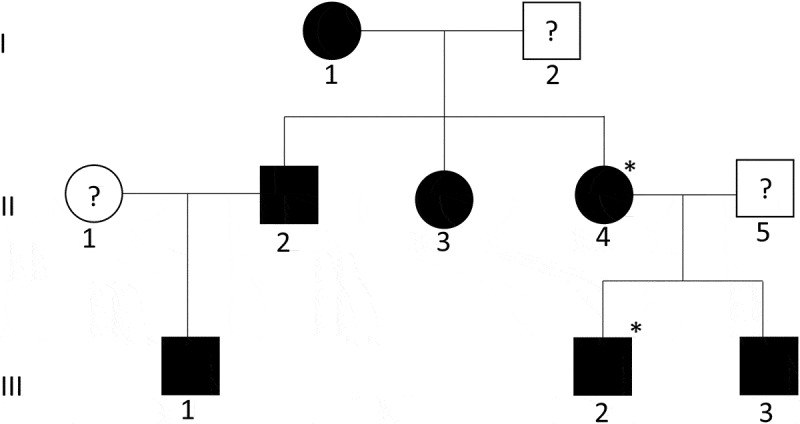
Identification of a *THBD* mutation in a family with a dominant form of inherited bleeding with normal platelet aggregation and secretion. Pedigree shows affected individuals as shaded symbols and asterisks (*) indicate patients (II:4 and III:2) whose whole exomes were sequenced.

The family pedigree in this study displays an autosomal dominant inheritance for the bleeding phenotype (Figure 1). An extensive bleeding history throughout the pedigree was evident. The index case, a male III:2, suffered from hemarthroses, soft tissue, and muscle bleeding in childhood and a spontaneous rectal sheath hematoma in 2006. His mother, II:4, was admitted to hospital for 3 weeks after wisdom teeth extraction and suffered bleeding after tonsillectomy and menorrhagia requiring hysterectomy (complicated by bleeding). His grandmother, I:1, suffered from bleeding after dental extraction requiring transfusion, easy bruising, and postpartum hemorrhage. His maternal aunt, II:3, had extensive bleeding which resorted in a nephrectomy following a road-traffic accident and maternal uncle II:2 lost an eye as a result of traumatic bleeding as a child as well as suffering from bleeding post dental extraction and soft tissue and muscle bleeding.

Patients’ III:2 and II:4 platelet aggregation traces showed normal responses to all the concentrations of agonists that were tested. Normal platelet granule secretion values were also observed in both patients in response to all agonists (data not shown). Therefore, no defect in platelet aggregation or secretion was detected in either of the patients tested.

WES identified 26 125 variants in II:4 and 25 969 variants in III:2. The bioinformatics pipeline was used to narrow down the variants with a series of filtering steps. Following the alignment step and determining the novelty of the variants, they were filtered against a custom-made panel of 856 genes known or predicted to be associated with platelet count or function, coagulation, or endothelial cell function. The remaining variants were then filtered by frequency; variants with a minor allele frequency (MAF) of more than or equal to 0.01 were excluded as were any synonymous changes. Comparison of both patients with a MAF ≤ 0.01 reduced to a total of 17 sequence variants. Pathogenicity prediction tools (Mutation Taster, Phastcons, SIFT, Provean etc.) were then utilized and the variants were classified as outlined by the American College of Medical Genetics and Genomics (ACMG) [9]. Candidate sequence variants were selected and reduced on the basis of a pathogenic prediction in at least three out of four of the prediction tools. This left candidate variants in five genes: *APC, PDE3A, ACE, OCLN*, and *THBD* (Table I). All previously mentioned variants were classified as being of “uncertain significance” by the ACMG except for *THBD* which was classified as “pathogenic.” Sanger sequencing confirmed that the genetic variant in *THBD* (c.1611C>A) was present in both patients.

**Table I.** Filtering pipeline showing the filtering strategy taken while selecting genetic variants as candidates in two affected family members (II:4 and III:2) with normal platelet function following extensive laboratory testing.

	II:4	III:2
Total number of variants identified by WES	26 125	25 969
Total number of variants (excluding synonymous) with a MAF ≤0.01	2320	2376
Shared significant variants from the panel of platelet and endothelial cell genes with a MAF ≤0.01	17	
Total number of novel variants	181	152
Total number of shared novel variants	50	
Total number of shared panel genes predicted to be pathogenic using all bioinformatics tools	5 (*APC, PDE3A, ACE, OCLN*, and *THBD*)	

## Discussion

Extensive lumiaggregometry performed in both patients established that there was no clear platelet function defect. The *THBD* sequence variant identified in this family which encodes the protein thrombomodulin (c.1611C>A, p.Cys537Stop) had been previously reported to cause a similar bleeding diathesis [10,11]. Thrombomodulin, a glycoprotein, is expressed on the surface of endothelial cells and normally serves as a cofactor for thrombin [12]. In a normal state, thrombomodulin forms a complex with thrombin reducing blood coagulation by activation of protein C, therefore inactivating factors Va and VIIIa, cofactors highly involved in the coagulation cascade, and promoting fibrinolysis. However, the genetic variant in these patients leads to a stop codon, resulting in premature termination of transcription and hence a truncated form of the protein. Due to the truncation, the protein is shed from the endothelial cell membrane into the blood plasma. The increase of soluble thrombomodulin promotes protein C activation and is also seen in several pathologic conditions associated with endothelial dysfunction [13,14]. This therefore reduces thrombin generation within a potential thrombus, resulting in the phenotype of posttraumatic bleeding [10]. Furthermore, this concurs with the abnormal prothrombin consumption index observed in the patients tested. This is a rare example of where a gain-of-function mutation in an anticoagulant factor causes a bleeding disorder [11].

## Conclusions

The genetic cause of bleeding in both patients I:2 and II:1 recruited to the GAPP study has been elucidated as a genetic variant in the gene *THBD* using next-generation sequencing. The same genetic variant in *THBD* has previously been shown to cause a bleeding phenotype within affected individuals [10,11]. This can, therefore, explain the cause of bleeding in II:4 and III:2 and suggests that it may be a recurrent mutation.

## References

[1] Bolton-Maggs PH, Chalmers EA, Collins PW, Harrison P, Kitchen S, Liesner RJ, Minford A, Mumford AD, Parapia LA, Perry DJ, et al A review of inherited platelet disorders with guidelines for their management on behalf of the UKHCDO. Br J Haematol 2006;135(5):603–633. 17107346 10.1111/j.1365-2141.2006.06343.x

[2] Nurden A, Nurden P. Advances in our understanding of the molecular basis of disorders of platelet function. J Thromb Haemost 2011;9(Suppl 1):76–91. 21781244 10.1111/j.1538-7836.2011.04274.x

[3] Leo VC, Morgan NV, Bem D, Jones ML, Lowe GC, Lordkipanidze M, Drake S, Simpson MA, Gissen P, Mumford A, et al Use of next-generation sequencing and candidate gene analysis to identify underlying defects in patients with inherited platelet function disorders. J Thromb Haemost 2015;13(4):643–650. 25556537 10.1111/jth.12836 PMC4383639

[4] Lowe GC, Lordkipanidze M, Watson SP. Utility of the ISTH bleeding assessment tool in predicting platelet defects in participants with suspected inherited platelet function disorders. J Thromb Haemost 2013;11(9):1663–1668. doi: 10.111/jth.12332. 23809206 PMC3773236

[5] Dawood BB, Lowe GC, Lordkipanidze M, Bem D, Daly ME, Makris M, Mumford A, Wilde JT, Watson SP. Evaluation of participants with suspected heritable platelet function disorders including recommendation and validation of a streamlined agonist panel. Blood 2012;120(25):5041–5049. 23002116 10.1182/blood-2012-07-444281 PMC3790949

[6] Johnson B, Lowe GC, Futterer J, Lordkipanidze M, MacDonald D, Simpson MA, Sanchez Guiu I, Drake S, Bem D, Leo V, et al Whole exome sequencing identifies genetic variants in inherited thrombocytopenia with secondary qualitative function defects Haematologica 2016;101(10):1170–1179. 10.3324/haematol.2016.146316 PMC5046646 27479822

[7] Lek M, Karczewski KJ, Minikel EV, Samocha KE, Banks E, Fennell T, O’Donnell-Luria AH, Ware JS, Hill AJ, Cummings BB, et al Analysis of protein-coding genetic variation in 60,706 humans. Nature 2016;536(7616):285–291. doi: 10.1038/nature19057. 27535533 PMC5018207

[8] Plagnol V, Curtis J, Epstein M, Mok KY, Stebbings E, Grigoriadou S, Wood NW, Hambleton S, Burns SO, Thrasher AJ, et al A robust model for read count data in exome sequencing experiments and implications for copy number variant calling. Bioinformatics (Oxford, England) 2012;28(21):2747–2754. 10.1093/bioinformatics/bts526 PMC3476336 22942019

[9] Richards S, Aziz N, Bale S, Bick D, Das S, Gastier-Foster J, Grody WW, Hegde M, Lyon E, Spector E, et al Standards and guidelines for the interpretation of sequence variants: a joint consensus recommendation of the American College of Medical Genetics and Genomics and the Association for Molecular Pathology. Genet Med 2015;17(5):405–424. 25741868 10.1038/gim.2015.30 PMC4544753

[10] Langdown J, Luddington RJ, Huntington JA, Baglin TP. A hereditary bleeding disorder resulting from a premature stop codon in thrombomodulin (p.Cys537Stop). Blood 2014;124(12):1951–1956. 25049278 10.1182/blood-2014-02-557538 PMC4168350

[11] Dargaud Y, Scoazec JY, Wielders SJH, Trzeciak C, Hackeng TM, Négrier C, Hemker HC, Lindhout T, Castoldi E. Characterization of an autosomal dominant bleeding disorder caused by a thrombomodulin mutation. Blood 2015;125(9):1497–1501. 25564403 10.1182/blood-2014-10-604553 PMC4342361

[12] Weiler H, Isermann BH. Thrombomodulin. J Thromb Haemost 2003;1(7):1515–1524. 12871287 10.1046/j.1538-7836.2003.00306.x

[13] Dahlback B, Villoutreix BO. The anticoagulant protein C pathway. FEBS Lett 2005;579(15):3310–3316. Epub 2005 Mar 13. 15943976 10.1016/j.febslet.2005.03.001

[14] Kurosawa S, DJ Stearns-Kurosawa, GT Kinasewitz Soluble thrombomodulin: a sign of bad times. Crit Care Med 2008;36(3):985–987. doi: 10.1097/CCM.0B013E318165FDA7. 18431290

